# Avoiding Plagiarism in Professional Writing

**Published:** 2012-05-01

**Authors:** Pamela Hallquist Viale

Writing for publication is an excellent way to communicate your ideas and knowledge to other readers. Publishing in the *Journal of the Advanced Practitioner in Oncology (JADPRO)* offers potential authors a venue to convey information in a variety of ways, including case studies, review articles on oncology subjects and advanced practice, commentary on unique issues affecting advanced practitioners, or discussions of new pharmaceutical agents. However, all potential authors must be sure that contributions to *JADPRO* or any publication contain accurate, well-referenced information and that the paper is written in a professional manner. Avoiding plagiarism is a critical part of the professional approach to writing for scientific publication.

## What Constitutes Plagiarism?

Plagiarism is the act of taking another's work or ideas and presenting them as your own (Cicutto, 2008). Although it may occur because of an author's lack of understanding regarding what constitutes plagiarism, it is a serious concern in medical publishing. The policy of the US Department of Health and Human Services Office of Research Integrity (ORI) is very clear: The ORI believes plagiarism is the theft or misappropriation of intellectual property and a significant copying of another's work without appropriate attribution (ORI, 2012).

There are several types of plagiarism. First, the most common form of plagiarism occurs when an author recreates sentences or paragraphs that are essentially identical to another's published work and does not acknowledge or reference the material (Das & Panjabi, 2011). Second, plagiarism of ideas may occur when an author takes an idea from someone else and passes it off as his or her own. An example of this might be an idea "stolen" from another professional presenting an idea at a conference or symposium (Das & Panjabi, 2011). In another scenario, a reviewer for a journal might read a paper that ends up not being accepted for publication. If the reviewer "adopts" the paper's main idea and publishes something similar on the topic, that is plagiarism (Das & Panjabi, 2011). A third form of plagiarism is self-plagiarism, in which an author publishes duplicate forms of his or her own originally published paper, containing redundant information or repeat study results (Cicutto, 2008).

While I was in nursing school, a fellow student submitted her thesis based on respiratory function; it was later discovered that she had essentially turned in an entire chapter from a well-respected nursing textbook. The professor excused the student partly based on the fact that a lack of understanding of publication ethics existed and that the plagiarism was unintentional; the professor also ruefully noted that she had given the chapter a grade of B-! The student had to submit a new paper, which might be considered an extremely forgiving action; consequences could have been more significant. The take-home message: All written work should be original and referenced appropriately (Das & Panjabi, 2011).

## Professional Writing

Professional writing for a scientific journal represents an implicit contract between the reader of the work and its author. It is expected that the author of the published paper is the sole writer responsible for the material; if additional information is included, the work must be referenced (Roig, 2011). It is accepted that authors writing a professional scientific paper will reference sentinel work or key papers as part of the foundation for an evidence-based paper on a given subject (Roig, 2011).

## Consequences of Plagiarism

Once discovered, plagiarism can have serious consequences. A journal can require its authors to notify his or her home institution of a plagiarism charge or publication infraction (Benos et al., 2005). If federal funding was a part of the publication, an inquiry is required by statute; clinical trials could be held until the outcome is determined (Benos et al., 2005). The ORI's webpage (ori.hhs.gov) lists updates on new misconduct findings by name of perpetrator, with a description of the misconduct. The list includes research misconduct as well as published papers and abstracts containing significant amounts of plagiarized text (ORI, 2012).

## Plagiarism Checkers

Although not a foolproof solution to the problem, a plagiarism checker can be a valuable tool for authors and editors alike. There are a number of these tools available online: some require a fee, whereas others are free for public use. Visit the *JADPRO* website at advancedpractitioner.com for a partial list of plagiarism checkers.

## In Closing

Authors must ensure that published work is original and referenced appropriately; sentences can be suspect if too close to the original material (Merriman, 2010). The Internet has made it all too easy to "cut and paste" another's published work into your own. Your new material should be rephrased and formatted to reflect your meaning while still referencing your support documents. If reproduction of information is needed in your manuscript, then permission must be sought from the publisher.

At *JADPRO*, we want to hear from you, and we look forward to publishing original papers from advanced practice authors.

## References

[1] Benos D. J., Fabres J., Farmer J., Gutierrez J. P., Hennessy K., Kosek D., Wang K. (2005). Ethics and scientific publication.. *Advances in Physiology Education*.

[2] Cicutto L. (2008). Plagiarism: Avoiding the peril in scientific writing.. *Chest*.

[3] Das N., Panjabi M. (2011). Plagiarism: Why is it such a big issue for medical writers?. *Perspectives in Clinical Research*.

[4] Merriman J. A. (2010). Plagiarism-What is it? How to avoid it.. *American Family Physician*.

[5] Office of Research Integrity. (2012). ORI policy on plagiarism.. http://www.ori.hhs.gov/ori-policy-plagiarism.

[6] Roig M. (2011). Avoiding plagiarism, self-plagiarism, and other questionable writing practices: A guide to ethical writing:. *Module.*.

